# Editorial: Biomarkers to disentangle the physiological from pathological brain aging, volume II

**DOI:** 10.3389/fnagi.2022.1088971

**Published:** 2022-11-22

**Authors:** Franca Rosa Guerini, Beatrice Arosio

**Affiliations:** ^1^IRCCS Fondazione Don Carlo Gnocchi ONLUS, Milan, Italy; ^2^Department of Clinical Sciences and Community Health, University of Milan, Milan, Italy

**Keywords:** neurodegenerative diseases, aging, biomarkers, bioimage analysis, comorbidities

The worldwide increase in human life expectancy, together with the concomitant rapid aging of the population represent phenomena that have a substantial impact on our societies, representing major challenges for public health. In this context dementia is one of the most common diseases related to aging. However, the clinical manifestation of neurodegenerative disorders occurs, in most cases, several years after the biological manifestation, when the clinical picture is already compromised. This Research Topic, which includes four original research papers and one systematic review, shed light on genetic, biochemical and bio-imaging assessments, by suggesting novel non-invasive tools for the early diagnosis of Alzheimer's Disease (AD) and to aid the diagnosis among different forms of dementia.

The first paper performed an extensive bioinformatics analysis of AD-associated gene expression profiles highlighting five immune-related hub genes (*CHGB, APLNR, FGF13, PAK1*, and *SERPINA3*) as promising prognostic and diagnostic markers for AD (Zhao et al.). Furthermore, competing endogenous ribonucleic acid network (mRNA–miRNA–lncRNA) and single Gene set enrichment analysis, conducted for each hub gene, evidenced that these genes were enriched in “oxidative phosphorylation” and, in particular, that the *AGAP3* gene is the most promising diagnostic target for AD development. Specifically, *AGAP3* was shown to exert a significant effect on AD by influencing immune infiltrating cells, especially CD4 cells, CD8 cells, and macrophages (Zhao et al.). The genetic approach shown in this study suggests future studies on immune-related genes providing new insight for the early diagnosis of AD.

Huang et al., based their study on the antioxidant role played by Uric Acid (UA) in oxidative stress, which seems to be directly involved in the pathological process of dementia (Butterfield and Halliwell, 2019). A positive correlation between the levels of UA in serum (sUA) and different values of Mini Mental Score Examination (MMSE) was reported in AD patients. Interestingly, the dynamic decline in sUA levels was correlated with the progression of cognitive impairment. Furthermore, sUA levels increased early in the AD process (i.e., mild cognitive impairment) and instead decreased in the overt disease, suggesting that sUA may be a protective factor of AD, especially in the advanced stage of dementia. Moreover, a positive correlation of sUA with plasma Aβ42 was observed in AD patients, but high levels of sUA were shown to alleviate both the CSF amyloid/tangle/neurodegeneration (ATN) biomarkers effects on cognitive impairments (by weakening their correlation with MMSE scores), and the correlation of CSF Aβ42 with tau.

The alteration in levels of Aβ42 and tau in CSF from AD occurs before the emergence of symptoms (Hansson et al., 2006). These alterations have also been suggested in various sleep disorders, including obstructive sleep apnea (OSA). Indeed, a systematic review and meta-analysis conducted by Cui et al., started from the hypothesis that OSA may be considered as possible precursor disorder triggering of the neurodegenerative process (Liguori et al., 2017; Sharma et al., 2018; Jorge et al., 2020; Diáz-Román et al., 2021). Decreased Aβ42 levels and increased total-tau levels in the CSF, and increased Aβ burden, by the means of PET imaging, were evidenced in OSA patients, especially in moderate/severe OSA. This evidence is of particular relevance in clinical practice, thanks to the fact that the existing therapies, able to reduce the severity of OSA, could relieve Aβ and tau burden delaying the progression of MCI to dementia.

MCI may convert into AD or other kinds of dementia as mixed dementia (MD) characterized, not only by neurodegenerative, but also by vascular findings (Alzheimer's Association, 2021). In this regard older persons with different forms of mild cognitive impairment and dementia were characterized both for the *VAMP2* gene polymorphism of 26bp insertion/deletion and for the VAMP2 mRNA expression (Costa et al.). VAMP2 is a vesicle protein of the SNARE complex that plays a crucial role in neural communication and plasticity (Weber et al., 1998). A higher frequency of subjects carrying the *VAMP2* 26bp Del polymorphism was reported in MD than in AD patients, and the highest VAMP2 mRNA expression was associated with this polymorphism. Notably, high levels of VAMP2 expression characterized MCI patients who later converted to MD. These results suggested that *VAMP2* 26 Del polymorphism and VAMP2 mRNA expression are possible early markers able to distinguish how the patient with mild cognitive impairment will evolve (MD or AD).

Finally a multimodal magnetic resonance imaging (MRI) approach was performed by Wu et al., to disentangle the brain normal aging from the asymptomatic phase of AD. Morphometry, functional connectivity, and tissue microstructure of hippocampal subfields were analyzed in cognitively normal and asymptomatic AD subjects clustered on the basis of the values obtained by the tau/Aβ42 ratio from CSF (cut off equal to 0.165).

In healthy aging as well as in dementia the morphometry, intrinsic functional connectivity, and tissue microstructure in all hippocampal subfields are disrupted. However, the subiculum and CA1-3 demonstrated the most robust correlations between imaging measures and the impairments of the neuropsychological performance that cause the onset of AD. Microstructural metrics from diffusion MRI are associated with neuropsychological assessments. Tau, rather than Aβ42, intimately correlates with synaptic and microstructural measures in hippocampal subfields. These results suggested hippocampal subfield connectivity and microstructural measures as promising imaging markers for early detection and prognosis of AD.

On the whole, this collection of articles suggests novel pathological mechanisms involved in neurodegenerative diseases and cognitive impairments. The evidences emerging from these studies are suggestive of a multidimensional approach in studying dementia starting from genes involved in oxidative stress pathway and/or in synaptic plasticity to correlated brain alterations. A more in-depth study in this sense will favor the development of useful tools for an early diagnosis of dementia, and for a preventive and more targeted therapeutic approach, allowing the growth of precision medicine-based treatments.

## Author contributions

Both authors listed have made a substantial, direct, and intellectual contribution to the work and approved it for publication.

## Conflict of interest

The authors declare that the research was conducted in the absence of any commercial or financial relationships that could be construed as a potential conflict of interest.

## Publisher's note

All claims expressed in this article are solely those of the authors and do not necessarily represent those of their affiliated organizations, or those of the publisher, the editors and the reviewers. Any product that may be evaluated in this article, or claim that may be made by its manufacturer, is not guaranteed or endorsed by the publisher.
